# In-Line Measurement of the Surface Texture of Rolls Using Long Slender Piezoresistive Microprobes

**DOI:** 10.3390/s21175955

**Published:** 2021-09-05

**Authors:** Linus Teir, Tuomas Lindstedt, Thomas Widmaier, Björn Hemming, Uwe Brand, Michael Fahrbach, Erwin Peiner, Antti Lassila

**Affiliations:** 1 VTT Technical Research Centre of Finland Ltd., National Metrology Institute VTT MIKES, 02150 Espoo, Finland; Bjorn.Hemming@vtt.fi (B.H.); Antti.Lassila@vtt.fi (A.L.); 2 RollResearch International Ltd., 02630 Espoo, Finland; tuomas.lindstedt@rollresearch.fi (T.L.); thomas.widmaier@rollresearch.fi (T.W.); 3 PTB, Physikalisch-Technische Bundesanstalt, 38116 Braunschweig, Germany; uwe.brand@ptb.de; 4 Laboratory for Emerging Nanometrology (LENA), Institut für Halbleitertechnik (IHT), Technische Universität Braunschweig, 38106 Braunschweig, Germany; m.fahrbach@tu-braunschweig.de (M.F.); e.peiner@tu-bs.de (E.P.)

**Keywords:** silicon microprobe, high speed, roughness, paper machine roll, metrology

## Abstract

Long slender piezoresistive silicon microprobes are a new type of sensor for measurement of surface roughness. Their advantage is the ability to measure at speeds of up to 15 mm/s, which is much faster than conventional stylus probes. The drawbacks are their small measurement range and tendency to break easily when deflected by more than the allowed range of 1 mm. In this article, previously developed microprobes were tested in the laboratory to evaluate their metrological properties, then tested under industrial conditions. There are several industrial measurement applications in which microprobes are useful. Measurement of the roughness of paper machine rolls was selected for testing in this study. The integration of a microprobe into an existing roll measurement device is presented together with the measurement results. The results are promising, indicating that measurements using a microprobe can give useful data on the grinding process.

## 1. Introduction

Surface roughness is an important feature for surfaces in contact, for example when mechanical components are in sliding or rolling contact. A poor surface roughness, in combination with load, speed and lubrication properties, can result in increased friction and wear. Surface roughness is also important in industries where the product is formed on rolls and is thus often measured in several of these industries [1]. Most often this is done using an inductive probe that measures a profile at speeds typically ranging from 0.5 mm/s to 1 mm/s [2]. In laboratory instruments, a translator linear guide creates a straight reference. In portable instruments, a skid close to the probe generates a reference as the skid slides across the highest peaks of the surface. Although this arrangement is far from ideal, most industrial roughness measurements are done with these affordable, portable instruments. Optical instruments based on, for example, coherence scanning interferometry (CSI) and focus variation are capable of measuring areal surface roughness and are becoming more popular, as a topographic map gives far more information on the surface structure than a profile does [3]. However, taking limitations of optical instruments [4,5] into account, contact probe instruments are still preferred by many industrial users. There is extensive ongoing research to reduce the impact of noise and environmental disturbances on optical instruments. Examples of this for CSI can be found in [6,7]. Even if the reliability issues with optical instruments were to be solved, they would probably remain expensive even in the future. In industrial applications and technical drawings, surface roughness is expressed by roughness parameters such as *Ra* and *Rz*, which are evaluated from the measured profile. These profile parameters are defined in the standard ISO 4287:1997 [8]. An overview of the most typical parameters of surface roughness used in manufacturing is given elsewhere [9].

Piezoresistive silicon microprobes have recently been developed for the fast measurement of surface roughness [10,11]. There are different probe sizes for different purposes, but generally the cantilever is a few millimetres long and several tenths of a millimetre wide. The microprobes are manufactured using silicon planar processing [12]. The sensing signal is obtained from piezoresistive strain gauges integrated into the cantilever near to the clamping point. The microprobes can be used in coordinate measuring machines, gear measuring machines, and instruments measuring surface texture [13]. When compared to traditional inductive surface-texture measurement probes, they have the advantage of providing roughness measurements at speeds of up to 15 mm/s [11]. Another advantage is their relatively low price compared to optical instruments for roughness measurements. The measurement range is about 200 µm, which is sufficient for the measurement of surface texture in the manufacturing industry. A disadvantage is potential breakage of the probe when the deflection surpasses its range of 1 mm [14,15]. Novel microprobe designs have improved wear resistance by using diamond tips [10] or hard coatings for the tip [16].

Rolls (large-scale cylindrical rotors in the paper and steel industry) are reground at regular intervals, and dimensional measurements are performed throughout the machining process [17,18]. Deviations from the required diameter, form and texture affect the quality of the end product. Therefore, roundness and cylindricity are measured during the grinding process, but not the texture. For the past two decades both were measured using a piece of equipment called a roll measuring device. An example of this equipment and its measurement uncertainty is described elsewhere [19,20]. In the rolling process the topography of the roll surface is reproduced on the end product. In some cases the human eye can detect stripes on the roll surface of just a few micrometres deviation, or even less. Surface roughness also plays an important role in the designed functionality of some rolls. For example, if the rolls are too smooth, the paper web can stick to them; if the friction is too low, the ability of the roll to transport the paper web suffers. Therefore, the possibility to measure form and roughness of the roll would provide useful feedback for the grinding process. The question is whether a microprobe would be suitable for these measurements or if it is too fragile for an industrial environment, or might be too sensitive to the typical disturbances in a workshop. This article examines the possibilities of using microprobes for the measurement of rolls and presents the results. Known issues with microprobes, such as tip wear and low damping, are beyond the scope of this paper.

## 2. Description of the Selected Microprobe Configuration

**Figure 1:**
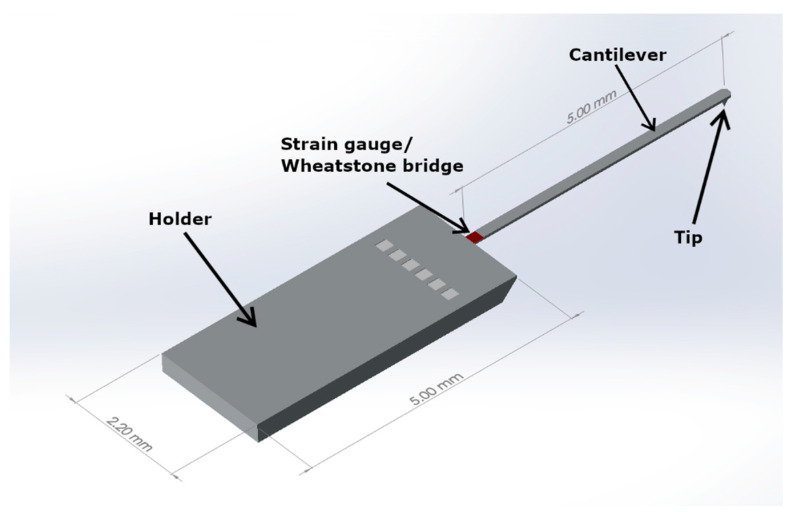
Illustration of a silicon microprobe.

Regular commercially available microprobes made of single crystal silicon were used in this study. These probes had no additional tip materials or coatings such as a diamond tip or aluminium oxide coating. The microprobes were produced by CiS Forschungsinstitut für Mikrosensorik GmbH (Erfurt, Germany) [21,22]. Their vital dimensions were the cantilever length of 5.0 mm and the shape and size of the tip; further structural dimensions are shown in Figure 1. The microprobe tip had an eight-sided pyramidal shape with a height of 100 µm. The radius of the microprobe tip was less than 2 µm with new sensors. The spring constant for the cantilever was 8.45 N/m [23].

When the cantilever was bent during measurement, the strain concentrated close to its connected base; four piezoresistive strain gauges were located there in a Wheatstone bridge configuration to enable measurement of the bending of the cantilever and displacement of the probe tip.

In order to get the best possible signal-to-noise ratio, a preamplifier for the Wheatstone bridge signal is needed as close to the probe as possible. In this study, a preamplifier electronics printed circuit board (PCB) design from Technische Universität Braunschweig [23] was redesigned for this purpose. It includes a low-noise voltage regulator for the Wheatstone bridge supply and an instrumentation amplifier for the bridge output voltage. The dimensions of the PCB were adjusted to a width of 50 mm and a length of 25 mm, which is better suited to the equipment intended for the industrial measurements. The bridge voltage was increased from 1 V to 3 V. The amplification gain was decreased to 61 to compensate for the voltage increase, reducing the noise amplification.

Frequency properties of the selected microprobe have been studied in earlier research [23,24], and the resonant frequency was calculated to be 2.8 kHz. Contact resonant frequency is much higher and slightly dependent on sample material, 9.6 to 16 kHz [24] and 14.1 to 14.3 kHz [23]. Using measurement speeds up to 10 mm/s, wavelengths down to 1 µm can be detected [24].

## 3. Measurement Setup in the Laboratory

**Figure 2:**
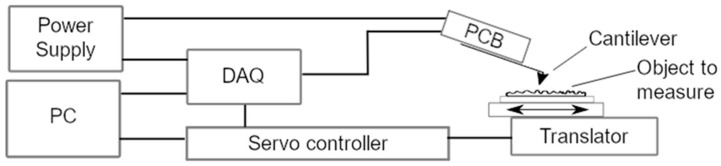
Schematic representation of the measurement setup. The translator, based on a piezoelectric drive, functions as a datum for the profile measurement and is controlled by the servo controller.

A microprobe setup was first built for laboratory use to test the electronics and data acquisition and to characterize the microprobe sensor. The setup is shown in Figure 2. The signal from the sensor is first preamplified on the PCB, then digitized with a NI-USB-6281 (National Instruments, Debrecen, Hungary) data acquisition card (DAQ). All data were transferred to a measurement software (NI Lab View 2019) running on a personal computer (PC), where they were recorded.

A different software (PIMikroMove 2.29.8.1) controlled the movement of the translator. The movement was created by an inertia drive, applying the driving force every 50 µs at a maximum speed of 10 mm/s.

**Figure 3:**
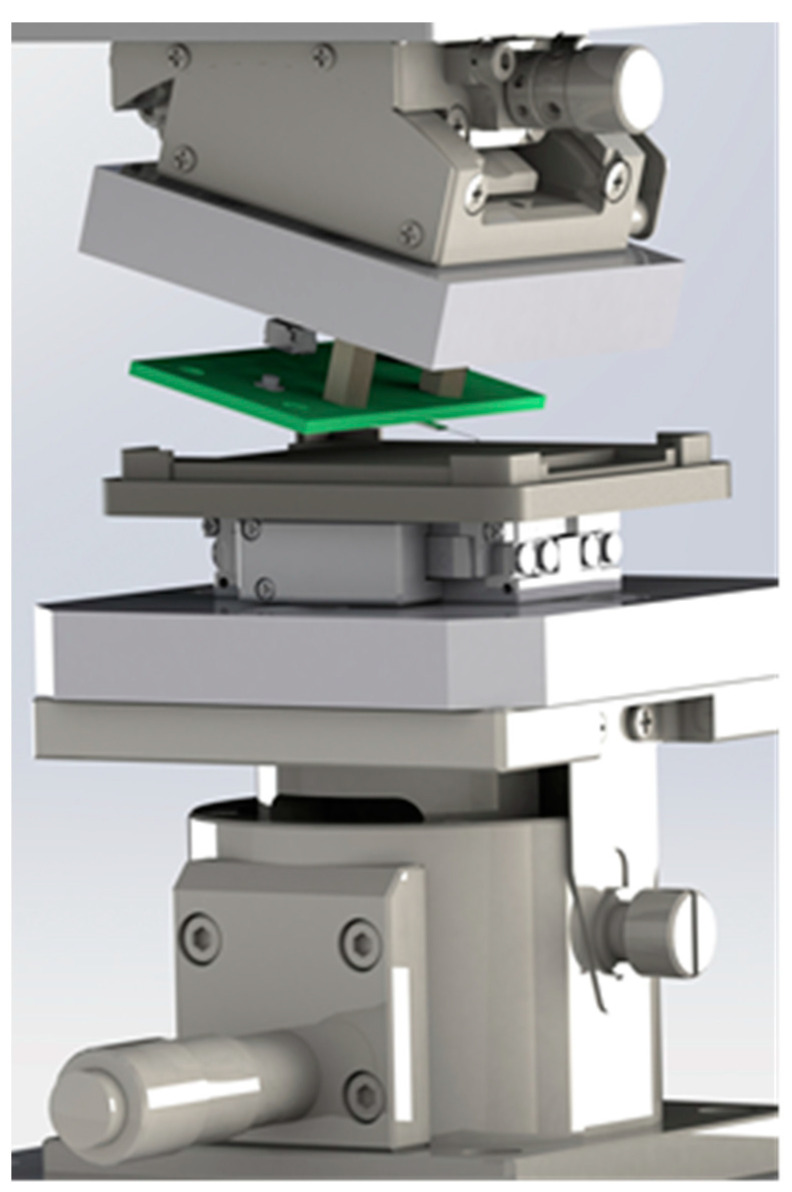
CAD design of the measurement setup. In green, the PCB with the microprobe can be seen.

The design of the measurement setup is shown in Figure 3 as a computer-aided design (CAD) render. The measurement setup features manual translation on the vertical *z*-axis and horizontal *x*-axis with manual linear stages, which allows optimal positioning of the piezoelectric linear drive relative to the microprobe.

## 4. Microprobe Sensor Set-Up for Roll Measurements

**Figure 4:**
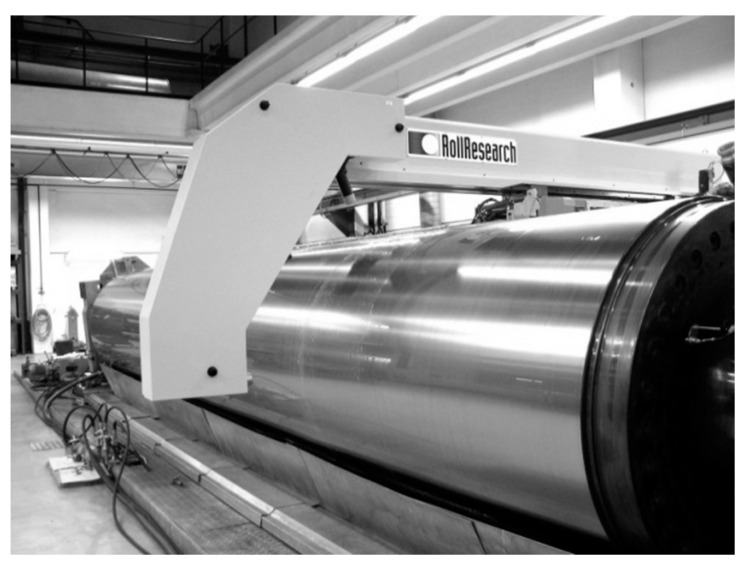
Roll measuring device for measuring the roundness and shape of a roll.

Roll grinding machines have for several decades been equipped with measuring devices (Figure 4) to measure the geometrical form of rolls [18]. For testing purposes, a microprobe was used to measure the local surface roughness profile of a roll from a paper machine. The measured roll was under overhaul and partially ground. The roll had a diameter of roughly 1 m, a length of roughly 8 m and was positioned in a grinding station with turning gear. The profile was measured parallel to the longitudinal axis of the roll.

Due to the fragility of the microprobes, a sliding skid was used in the tests to protect the microprobe from deflections that were too high. The skid also worked as a reference or datum for the measured profile. The roll was cylindrical, and the skid had the shape of a plane, giving a cylinder/plane contact. The sliding skid was sufficient for measuring roughness and acceptable for measuring waviness to within a few millimetres. The integration of the microprobe into the roll measuring device is described in detail in an earlier thesis [25].

**Figure 5:**
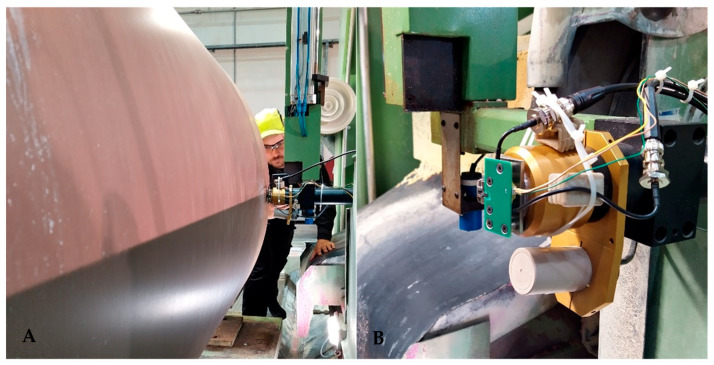
Paper roll with microprobe in the background (**A**). The roll measuring device in the picture (**B**) is of a different type to the one illustrated in Figure 7 and described in the text, but the head is similar.

A microprobe holder was designed specifically for this study to enable mechanical installation of the microprobe on a roll measuring device. The microprobe was mounted on one of the measuring probes of the existing roll geometry device as shown in Figure 5. This allows the diameter variation or alignment errors of the roll to be ignored during roughness measurements, as the measuring probe moves radially relative to the roll. Moreover, the motion axes of the grinding machine can be used to perform roughness measurements. The microprobe was placed in contact with the roll on an axis moving radially relative to the roll. Measurements were performed on an axis moving parallel to the longitudinal axis of the roll. The measuring process was carried out in the same sequence each time: first make contact, then start the movement along the longitudinal axis of the roll.

During this study, the power supply and data acquisition setup was the same as in the laboratory. However, the power for the microprobe could be drawn from the grinding machine’s own power supply, and the data could be collected, processed and stored using the existing PC components of the grinding machine.

**Figure 6:**
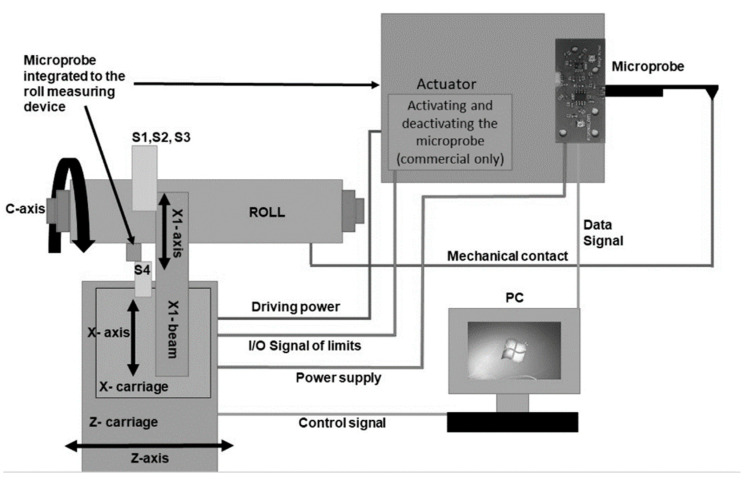
Schematic representation of microprobe integration into a roll measuring device. The microprobe is mounted on a measuring probe (S4) and is in mechanical contact with the roll. Power is supplied from the grinding machine, and the data are processed with and stored on a PC.

**Figure 7:**
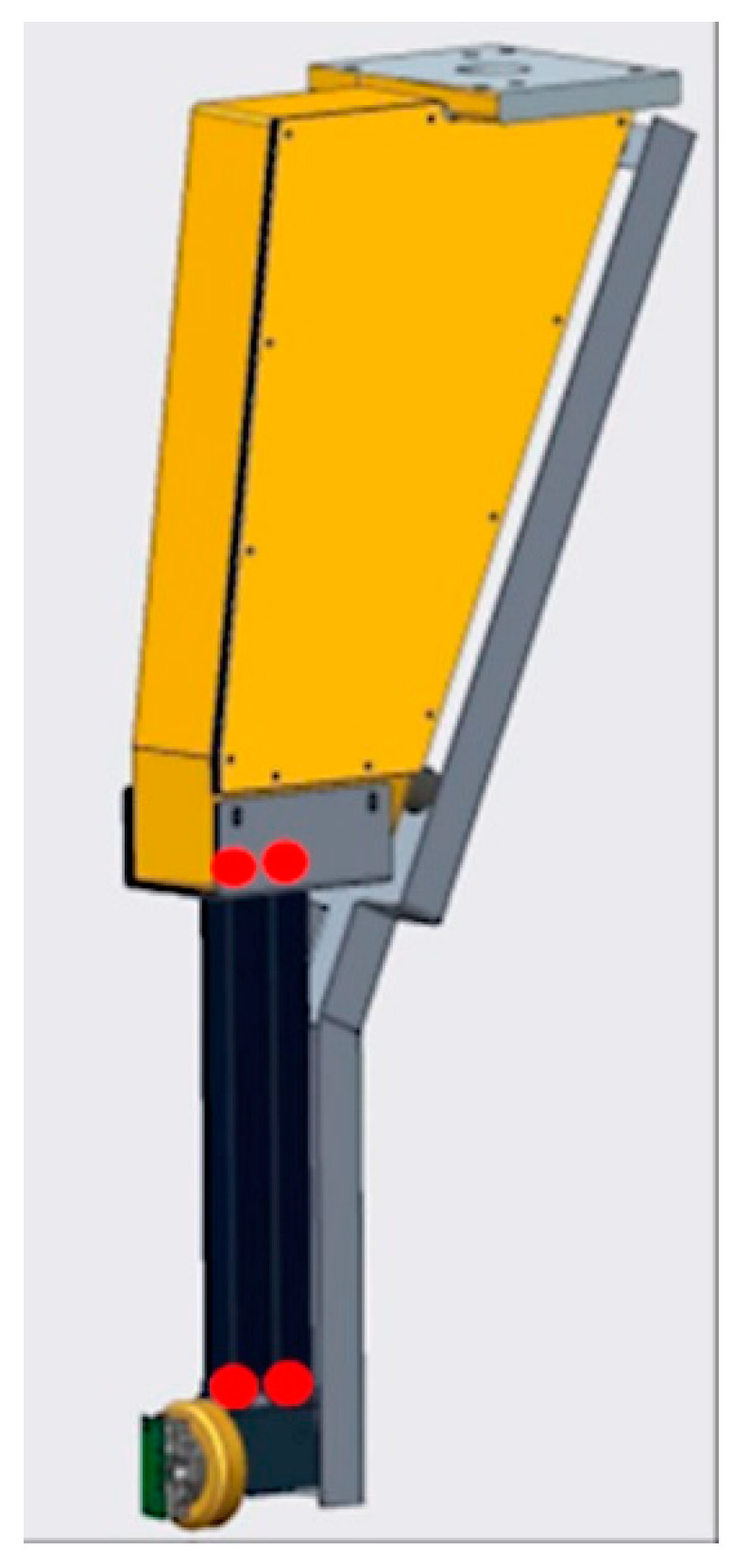
CAD design of microprobe integration in a roll measuring instrument.

Figure 6 shows a planned schematic integration of the microprobe into a grinding machine, including future improvements for a commercial version. The microprobe was mounted on a part of the roundness measuring instrument called the S4 arm. Figure 7 shows the S4 arm and the microprobe with the holder, which replaces the original measuring head of the arm. The kinematics are based on four bar linkages containing a spring, which pushed the microprobe with skid into contact with the roll. With this mechanism the orientation of the probe does not change when moved into contact. The four pivot points of the arm allow filtering out of the diameter variation of the roll and are indicated in red in Figure 7.

**Figure 8:**
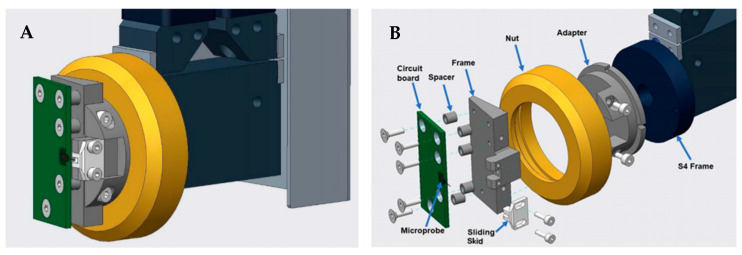
CAD design (**A**) and exploded view (**B**) of microprobe integration in a roll measuring instrument.

Figure 8 shows the mounted microprobe and holder design on the left and an exploded view of the holder assembly on the right. The assembly consists of five individual parts and a nut, which belongs to the original S4 design. The parts are named in Figure 8. When the microprobe was mounted, there was a 12-degree angle between the roll surface and the microprobe.

With the microprobe integration used in this study, the diameter range of the rolls that can be measured was 300 mm to 2000 mm. Measuring length is not limited by the integration method. However, the tip durability of the microprobe is limited, and the size of the grinding machine determines the longitudinal travel along the roll.

## 5. Laboratory Characterization of the Microprobe Sensor

Characterization of the microprobe followed the guidelines presented in standards ISO 25178-601 [26] and ISO 3274 [27]. These included measurement of an optical flat, both tilted and horizontally aligned, measurement of a roughness standard with sinusoidal profile, and a static contact test. In addition, a free-hanging test was done to check noise without tip contact to a sample. The tests are described in detail elsewhere [28] and are only briefly depicted here.

First, the sensitivity was determined using a depth setting standard. The standard was of type A according to the classification in ISO 5436-1 [29]. The standard manufactured by Halle Präzisions-Kalibriernormale GmbH (Edemissen, Germany) has six grooves of depth in the range from 0.3 µm to 8.6 µm. As the depths are small compared to the measuring range of the microprobe, the standard was measured at three offset heights within the measuring range of the probe. Resulting from the sensitivity evaluation, a linearity error of about ± 0.8% was calculated when comparing the results from the three offset heights.

**Figure 9:**
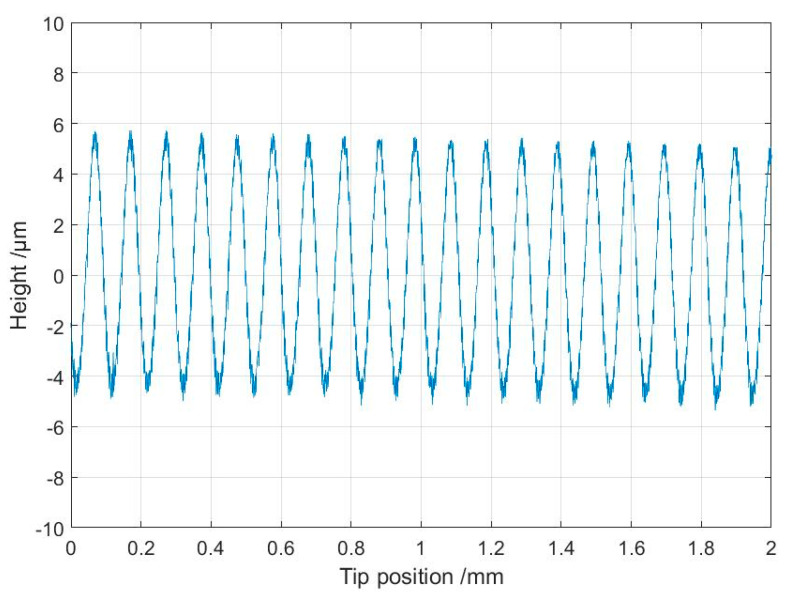
Partial section of the measurement of type 178-601 surface roughness standard using the microprobe setup.

With this calibration, the measurement setup was able to produce profile measurements with a vertical scale in length units. The next standard to be used was of type C according to ISO 5436-1 [29], with a sine-wave surface profile. A standard manufactured by Mitutoyo (type 178-601, S/N 131883) was selected. The standard was calibrated using the Taylor Hobson Talysurf 2 reference instrument equipped with an inductive stylus probe at VTT MIKES. The traceability for this reference instrument is described elsewhere [30]. In Table 1, the results of the selected ISO 4287 [8] parameters measured using the microprobe setup are compared with the calibrated values. A measured profile using the microprobe setup is shown in Figure 9. The measurement correlated well with the calibration values, as the deviations were less than the uncertainties of the calibrated values. When comparing the results from different measurements, it should be noted that there is always some inhomogeneity in roughness standards. With a stylus instrument the measurements would be time consuming if the complete area were covered with thousands of traced profiles. However, the inhomogeneity of the standard was thoroughly checked using the Talysurf (Leicester, UK) reference instrument. Using one single sampling length (cut-off) of 2.5 mm, the spread was ±1.3% for *Ra* and ±6.3% for the *Rz* parameter. As the evaluation length included several lengths, this variation will be reduced but cannot be completely ignored.

A free-hanging tip characterization test of the microprobe resulted in good stability; the standard deviation of the microprobe output corresponded to 4 nm, during an eight-minute-long measurement with a sample rate of 10,000 samples per second in good laboratory conditions. Measurement of an inclined flat at a scanning speed of 2 mm/s resulted in a standard deviation of the microprobe output of 140 nm with a sample rate of 100,000 samples per second.

## 6. Results of Industrial Test Measurements

**Figure 10:**
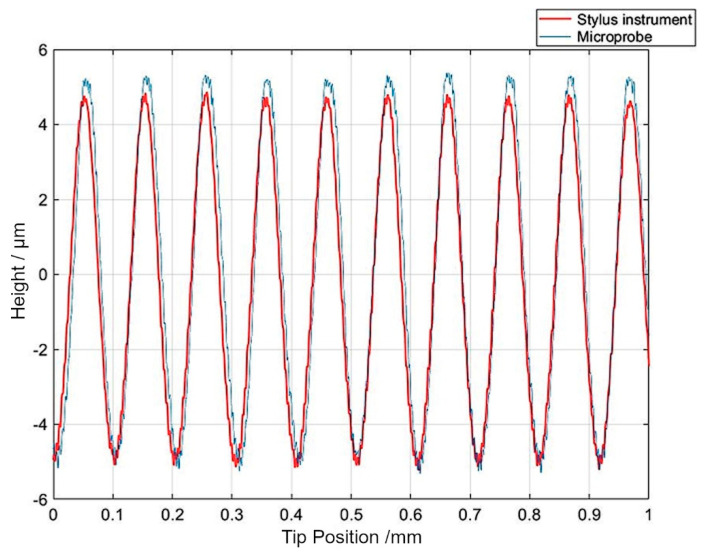
Comparison of the primary profiles of a Mitutoyo 178-601 (S/N 131883) measured with the reference instrument (Talysurf) and with the industrial microprobe setup (at 1.67 mm/s and sampling rate 100 kHz).

The tests at the industrial site consisted of measurements of roughness standards and measurements of the roll. The results obtained with the Mitutoyo 178-601 roughness standard are presented here. The raw data from the Mitutoyo 178-601 (S/N 131883), measured with the industrial microprobe setup at a scanning speed of 1.67 mm/s and a data acquisition rate of 100 kHz, showed high-frequency fluctuations at wavelengths ranging from roughly 1 µm to 5 µm and amplitudes ranging from 0.2 µm to 0.4 µm. The data measured in the laboratory with the stylus reference instrument (Talysurf) (Leicester, UK) also showed a wavelength component of roughly 3.5 to 5 µm and an amplitude up to 0.4 µm. It can be assumed that the roughness standard contains some short wavelengths originating from its manufacturing process and that higher fluctuations measured by the microprobe are caused by its mechanical and electrical properties in the industrial environment. In surface metrology, wavelengths close to the tip dimensions are filtered out by the *λs* filter to get the primary profile defined in ISO 4287:1997 [8]. The cut-off for the *λs* filter was selected at 2.5 µm, although ISO 3274:1996 [27] specifies 8 µm as the default, which would remove even more high-frequency content. Figure 10 shows a comparison of primary profiles measured with the stylus instrument and the microprobe. From the data, selected roughness parameters were calculated using the Mountain Map 6 software. Table 2 compares the calibrated values. The variation of the industrial microprobe measurements in Table 2 was about 8%.

One purpose of the tests in an industrial environment was to investigate the influence of acoustic, mechanical or electrical disturbances on microprobe operation. During two consecutive measurements with the Mitutoyo 178-601, standard background noise levels during static contact were recorded before, between and after the measurements. The standard deviation of the noise before the measurement was 54 nm over 2 s. Between the measurements the standard deviation of the noise was 155 nm over 4 s. After both measurements the standard deviation of the noise was 157 nm over 16 s. In comparison, in measurements under laboratory conditions the standard deviation of noise during static contact was under 10 nm [28].

**Figure 11:**
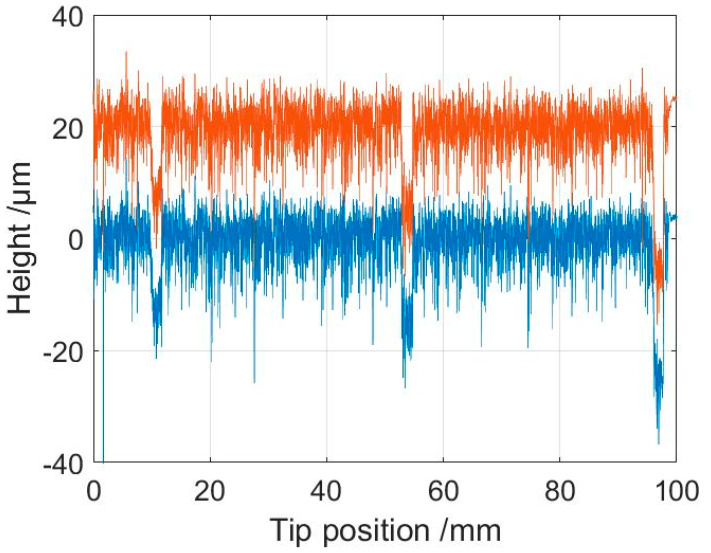
Two consecutive measured profiles of a roll measured at 1.67 mm/s and 100 kHz sampling rate. One profile is shifted by an offset of 20 µm.

**Figure 12:**
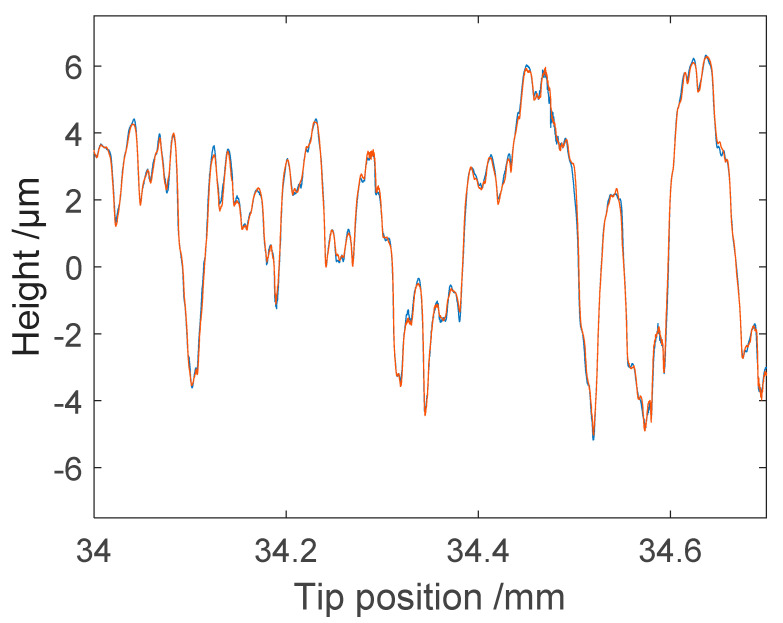
Enlarged detail of Figure 11 showing the repeatability of two consecutive measured profiles.

**Figure 13:**
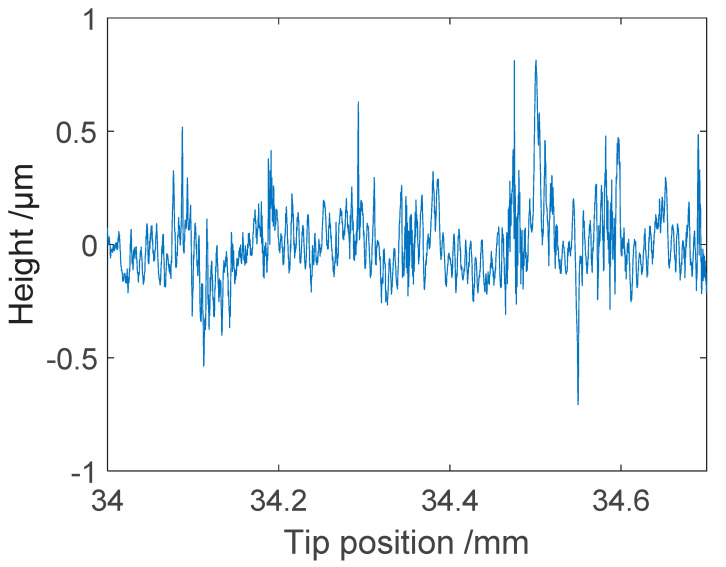
Difference between two consecutive measured profiles.

To study the repeatability of the microprobe system in measuring the roll topography, consecutive profiles were measured, as shown in Figure 11. Figure 12 shows partial signals on an enlarged x-scale. As seen in Figure 13, two consecutive profiles generally differed by less than 0.2 µm. This indicates that the microprobe sensor gave accurate, repeatable information on roll topography.

**Figure 14:**
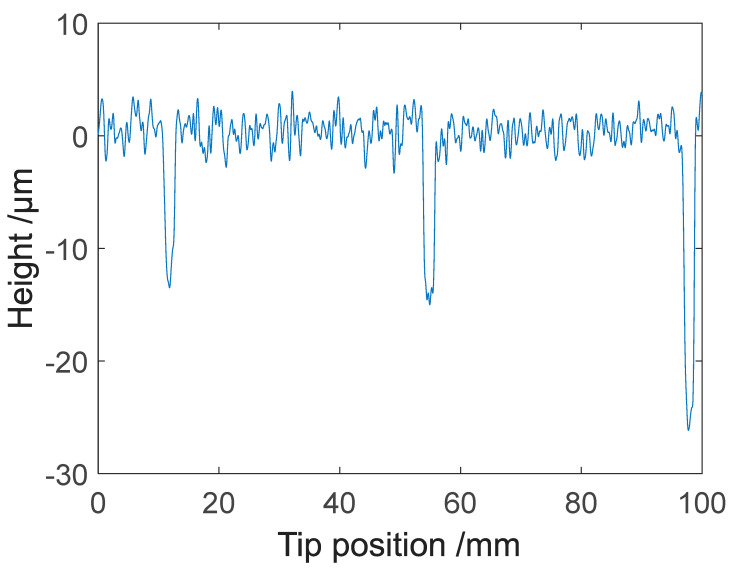
Waviness profile with Gaussian filter and 0.8 mm cut off. Three valleys with depths over 10 µm are visible.

As the microprobe is equipped with a sliding skid (see Figure 8), it has limitations regarding measurements of waviness. However, for the purpose of feedback for the grinding process it might be useful to perform a waviness analysis. In Figure 14, short wavelengths are filtered out using a cut-off wavelength of 0.8 mm. This cut-off wavelength is small compared to the dimensions of the sliding skid. Three large repeated valleys are now visible in the profiles, which tells us that there is a small mismatch between the dimensions of the grinding wheel and the pitch control of its movement. This is an additional example of useful data provided by microprobe measurements. Applying the spring constant to the measurement deflection from Section 2, we estimate the measurement force to be close to 0.5 mN for results presented in this section.

## 7. Conclusions

Microprobes make surface roughness measurements possible at relatively low cost, and they have several advantages. The conclusion is summarized in the following.

To the knowledge of the authors this is the first time that a microprobe for roughness measurement has been developed for integration into a grinding machine. The advantages are small size, relatively low price and easy integration into the roll measuring instrument.

A microprobe measurement sensor with test set-up has been designed, built and characterized. The printed circuit board is based on a previous design, but the microprobe was not altered. Using the test setup, measurements were performed characterizing the sensitivity, noise and linearity at scanning speeds up to 1.67 mm/s. Integration of the microprobe sensor into a roll measuring machine is presented, together with test results.

The ability of the microprobe sensor to measure surface roughness was verified by measuring a traceably calibrated surface roughness standard (*Ra*, *Rz* and *RSm* of ≈3 µm, ≈10 µm and ≈100 µm, respectively). The comparison of primary profiles measured with the stylus instrument and microprobe showed good agreement, considering that there are uncertainties in finding the same location of the profile combined with the inhomogeneity of the roughness standard (Figure 10). Tests performed in the laboratory and in an industrial setting showed measurement deviations of less than 6% and less than 3%, respectively, for selected parameters.

A typical uncertainty in the calibration of a roughness standard at the National Standards Laboratories and accredited laboratories is 4–5% [2]. When roughness standards are used in workshops for calibration of a measurement instrument, the uncertainty level is up to 8% [2]. The relative deviation of the industrial measurement’s amplitude parameters (presented in Table 2) was about 2%, which is good for an industrial device and can be considered excellent for an initial result.

Although not perfect, the initial results are acceptable and promising. The microprobe also shows good potential for other industries in which quick and low-cost roughness measurements are needed.

However, further development and testing will be needed before the presented industrial application becomes commercially available. One practical challenge is that the mechanical design of the sliding shoe should protect the microprobe in almost all situations. In case of broken probe, it should be easy to install a new probe.

A topic for future research in the field of mechanical engineering and paper manufacture would be to investigate the roughness parameters and magnitude, which are relevant to end users in the paper industry and steel industry. This is related to measurement strategy including point density, filtering and the number and lengths of profiles to be measured.

## Figures and Tables

**Table 1:** Comparison of *Ra*, *Rz* and *RSm* parameters for a type 178-601 surface roughness standard measured with the Talysurf and the microprobe setup in the laboratory.

Parameter	Reference Value by Talysurf µm	Uncertainty of Reference Value (k = 2) µm	Measured with Microprobe µm	Deviation µm	Relative Deviation %
*Ra*	3.07	0.31	2.89	−0.18	−5.9
*Rz*	9.80	1.47	9.97	0.17	1.7
*RSm*	101.47	10.15	101.60	0.13	0.1

**Table 2:** Comparison of average *Ra*, *Rz* and *RSm* parameters measured with Talysurf and an industrial microprobe setup.

Parameter	Reference Value by Talysurf	Uncertainty of Reference Value (k = 2)	Measured with Microprobe	Deviation	Relative Deviation
	µm	µm	µm	µm	%
*Ra*	3.07	0.31	2.99	−0.08	−2.6
*Rz*	9.80	1.47	9.71	−0.09	−0.92
*RSm*	101.47	10.15	101.78	−0.36	−0.35

## Data Availability

The data will be made available on zenodo.org.

## Sample Availability

Samples of the compounds are not available from the authors.
